# Supplementary material for: Modeling glioblastoma heterogeneity as a dynamic network of cell states
Source: Mol Syst Biol. 2021 Sep 16;17(9):e10105. doi: 10.15252/msb.202010105 (PMC8444284; doi:10.15252/msb.202010105)
Supplement: Supplementary file 6 — Source Data for Figure 5 [file MSB-17-e10105-s004.zip › Figure5A_sourcedata/GSEA_3071/nosigdb_stateI.GseaPreranked.1622795145664/SINGH_SOX2_UPREGULATED_TRANSCRIPTS_PMID_28122245.html]

Details for gene set SINGH\_SOX2\_UPREGULATED\_TRANSCRIPTS\_PMID\_28122245[GSEA]

|  || Dataset | state13071 |
| Phenotype | NoPhenotypeAvailable |
| Upregulated in class | na\_neg |
| GeneSet | SINGH\_SOX2\_UPREGULATED\_TRANSCRIPTS\_PMID\_28122245 |
| Enrichment Score (ES) | -0.55475485 |
| Normalized Enrichment Score (NES) | -3.2046752 |
| Nominal p-value | 0.0 |
| FDR q-value | 0.0 |
| FWER p-Value | 0.0 |
Table: GSEA Results Summary

  

Fig 1: Enrichment plot: SINGH\_SOX2\_UPREGULATED\_TRANSCRIPTS\_PMID\_28122245      
 Profile of the Running ES Score & Positions of GeneSet Members on the Rank Ordered List

  

| PROBE | GENE SYMBOL | GENE\_TITLE | RANK IN GENE LIST | RANK METRIC SCORE | RUNNING ES | CORE ENRICHMENT || 1 | PTCH1 |  |  | 7 | 1.951 | 0.0121 | No |
| 2 | TLE3 |  |  | 126 | 0.968 | -0.0163 | No |
| 3 | TMEM100 |  |  | 170 | 0.872 | -0.0229 | No |
| 4 | LFNG |  |  | 228 | 0.772 | -0.0344 | No |
| 5 | VAV3 |  |  | 325 | 0.650 | -0.0586 | No |
| 6 | PSAT1 |  |  | 338 | 0.638 | -0.0575 | No |
| 7 | MAGED1 |  |  | 391 | 0.599 | -0.0688 | No |
| 8 | TJP1 |  |  | 557 | 0.477 | -0.1149 | No |
| 9 | ARHGAP42 |  |  | 733 | 0.402 | -0.1646 | No |
| 10 | JAG1 |  |  | 796 | 0.383 | -0.1805 | No |
| 11 | HMGCR |  |  | 875 | 0.357 | -0.2013 | No |
| 12 | HMGCS1 |  |  | 994 | 0.328 | -0.2344 | No |
| 13 | ZNF521 |  |  | 1005 | 0.327 | -0.2350 | No |
| 14 | LIX1L |  |  | 1057 | 0.316 | -0.2481 | No |
| 15 | NR3C1 |  |  | 1162 | 0.297 | -0.2772 | No |
| 16 | CTNND1 |  |  | 1338 | 0.261 | -0.3279 | No |
| 17 | CCDC34 |  |  | 1376 | 0.255 | -0.3371 | No |
| 18 | FAM192A |  |  | 1419 | -0.250 | -0.3480 | No |
| 19 | NDUFA6 |  |  | 1420 | -0.250 | -0.3461 | No |
| 20 | NHSL1 |  |  | 1421 | -0.250 | -0.3443 | No |
| 21 | ARMC6 |  |  | 1480 | -0.255 | -0.3599 | No |
| 22 | AKIRIN2 |  |  | 1507 | -0.258 | -0.3658 | No |
| 23 | KAT6A |  |  | 1558 | -0.263 | -0.3789 | No |
| 24 | SEC22C |  |  | 1562 | -0.263 | -0.3779 | No |
| 25 | RIMKLB |  |  | 1567 | -0.263 | -0.3772 | No |
| 26 | HIST1H3G |  |  | 1597 | -0.267 | -0.3840 | No |
| 27 | SPRY2 |  |  | 1612 | -0.269 | -0.3862 | No |
| 28 | IPO9 |  |  | 1634 | -0.272 | -0.3906 | No |
| 29 | PPAT |  |  | 1655 | -0.275 | -0.3946 | No |
| 30 | THUMPD3 |  |  | 1671 | -0.276 | -0.3971 | No |
| 31 | DENND2A |  |  | 1699 | -0.279 | -0.4032 | No |
| 32 | NPAS3 |  |  | 1708 | -0.281 | -0.4035 | No |
| 33 | ACD |  |  | 1713 | -0.282 | -0.4027 | No |
| 34 | RPA1 |  |  | 1717 | -0.282 | -0.4015 | No |
| 35 | EDNRB |  |  | 1754 | -0.287 | -0.4103 | No |
| 36 | SMARCA4 |  |  | 1762 | -0.289 | -0.4103 | No |
| 37 | DPF3 |  |  | 1767 | -0.290 | -0.4094 | No |
| 38 | DDAH1 |  |  | 1791 | -0.292 | -0.4141 | No |
| 39 | ELP3 |  |  | 1856 | -0.300 | -0.4312 | No |
| 40 | PLCE1 |  |  | 1890 | -0.303 | -0.4389 | No |
| 41 | CHIC2 |  |  | 1910 | -0.307 | -0.4424 | No |
| 42 | KPNA2 |  |  | 1944 | -0.312 | -0.4500 | No |
| 43 | ZBTB10 |  |  | 1956 | -0.313 | -0.4511 | No |
| 44 | CEP78 |  |  | 2000 | -0.317 | -0.4617 | No |
| 45 | POU2F1 |  |  | 2010 | -0.318 | -0.4621 | No |
| 46 | OPA1 |  |  | 2014 | -0.319 | -0.4607 | No |
| 47 | SOX6 |  |  | 2032 | -0.323 | -0.4634 | No |
| 48 | OLIG2 |  |  | 2044 | -0.324 | -0.4644 | No |
| 49 | HIST1H2AG |  |  | 2090 | -0.333 | -0.4755 | No |
| 50 | SP4 |  |  | 2110 | -0.336 | -0.4787 | No |
| 51 | NDE1 |  |  | 2111 | -0.337 | -0.4763 | No |
| 52 | RNF157 |  |  | 2160 | -0.345 | -0.4882 | No |
| 53 | KIAA1958 |  |  | 2170 | -0.347 | -0.4884 | No |
| 54 | ADK |  |  | 2186 | -0.349 | -0.4903 | No |
| 55 | DCPS |  |  | 2194 | -0.351 | -0.4899 | No |
| 56 | GLCCI1 |  |  | 2212 | -0.356 | -0.4924 | No |
| 57 | CENPA |  |  | 2220 | -0.357 | -0.4919 | No |
| 58 | DHX36 |  |  | 2226 | -0.359 | -0.4908 | No |
| 59 | TRIL |  |  | 2250 | -0.363 | -0.4951 | No |
| 60 | STIM2 |  |  | 2256 | -0.364 | -0.4939 | No |
| 61 | FAM84B |  |  | 2258 | -0.365 | -0.4916 | No |
| 62 | SMAD1 |  |  | 2265 | -0.365 | -0.4907 | No |
| 63 | NUCKS1 |  |  | 2278 | -0.368 | -0.4916 | No |
| 64 | MTHFD1 |  |  | 2282 | -0.369 | -0.4899 | No |
| 65 | SCD5 |  |  | 2299 | -0.373 | -0.4920 | No |
| 66 | DUSP16 |  |  | 2309 | -0.374 | -0.4919 | No |
| 67 | MSI2 |  |  | 2314 | -0.375 | -0.4904 | No |
| 68 | TRAF4 |  |  | 2321 | -0.376 | -0.4895 | No |
| 69 | CEP128 |  |  | 2323 | -0.377 | -0.4870 | No |
| 70 | ETV4 |  |  | 2325 | -0.378 | -0.4846 | No |
| 71 | PCCB |  |  | 2343 | -0.382 | -0.4869 | No |
| 72 | PAG1 |  |  | 2347 | -0.383 | -0.4850 | No |
| 73 | VPS37B |  |  | 2363 | -0.386 | -0.4867 | No |
| 74 | PTOV1 |  |  | 2375 | -0.388 | -0.4872 | No |
| 75 | EXOSC2 |  |  | 2400 | -0.392 | -0.4916 | No |
| 76 | BUB1 |  |  | 2416 | -0.396 | -0.4932 | No |
| 77 | ARHGEF26 |  |  | 2465 | -0.404 | -0.5047 | No |
| 78 | FIGN |  |  | 2543 | -0.426 | -0.5247 | No |
| 79 | PAICS |  |  | 2545 | -0.426 | -0.5219 | No |
| 80 | APC |  |  | 2550 | -0.427 | -0.5200 | No |
| 81 | CDK5RAP2 |  |  | 2564 | -0.431 | -0.5208 | No |
| 82 | HIST1H2AL |  |  | 2582 | -0.435 | -0.5227 | No |
| 83 | TMEM106C |  |  | 2592 | -0.437 | -0.5223 | No |
| 84 | RMI1 |  |  | 2616 | -0.444 | -0.5259 | No |
| 85 | SUGP2 |  |  | 2650 | -0.454 | -0.5326 | No |
| 86 | SKA2 |  |  | 2672 | -0.464 | -0.5355 | No |
| 87 | CTNND2 |  |  | 2678 | -0.465 | -0.5336 | No |
| 88 | FOXO3 |  |  | 2696 | -0.471 | -0.5353 | No |
| 89 | PHLPP1 |  |  | 2717 | -0.475 | -0.5378 | No |
| 90 | HIST1H2BH |  |  | 2748 | -0.485 | -0.5433 | No |
| 91 | TUBA1A |  |  | 2771 | -0.495 | -0.5463 | No |
| 92 | HIST1H3D |  |  | 2790 | -0.499 | -0.5481 | No |
| 93 | DCAF16 |  |  | 2801 | -0.501 | -0.5475 | No |
| 94 | WDR82 |  |  | 2807 | -0.503 | -0.5453 | No |
| 95 | DOK5 |  |  | 2813 | -0.507 | -0.5431 | No |
| 96 | LSM5 |  |  | 2820 | -0.509 | -0.5412 | No |
| 97 | ITGB8 |  |  | 2840 | -0.516 | -0.5432 | No |
| 98 | RAD21 |  |  | 2866 | -0.527 | -0.5468 | No |
| 99 | ANP32E |  |  | 2877 | -0.530 | -0.5460 | No |
| 100 | SPATA6 |  |  | 2882 | -0.531 | -0.5433 | No |
| 101 | ELOVL2 |  |  | 2891 | -0.535 | -0.5418 | No |
| 102 | SPECC1 |  |  | 2909 | -0.546 | -0.5430 | No |
| 103 | HNRNPUL1 |  |  | 2913 | -0.549 | -0.5399 | No |
| 104 | CMSS1 |  |  | 2931 | -0.561 | -0.5409 | No |
| 105 | PBX3 |  |  | 2959 | -0.578 | -0.5448 | No |
| 106 | CELSR2 |  |  | 2993 | -0.597 | -0.5504 | Yes |
| 107 | TPX2 |  |  | 3006 | -0.606 | -0.5496 | Yes |
| 108 | SOX21 |  |  | 3011 | -0.608 | -0.5463 | Yes |
| 109 | NOVA1 |  |  | 3040 | -0.627 | -0.5502 | Yes |
| 110 | OLIG1 |  |  | 3055 | -0.638 | -0.5497 | Yes |
| 111 | AURKB |  |  | 3056 | -0.639 | -0.5451 | Yes |
| 112 | BCL7A |  |  | 3058 | -0.641 | -0.5407 | Yes |
| 113 | CDKN1B |  |  | 3065 | -0.646 | -0.5378 | Yes |
| 114 | FANCI |  |  | 3074 | -0.656 | -0.5354 | Yes |
| 115 | CDC45 |  |  | 3081 | -0.661 | -0.5324 | Yes |
| 116 | TMPO |  |  | 3088 | -0.666 | -0.5294 | Yes |
| 117 | MAD2L1 |  |  | 3099 | -0.675 | -0.5275 | Yes |
| 118 | OSBPL6 |  |  | 3102 | -0.679 | -0.5231 | Yes |
| 119 | LSM4 |  |  | 3115 | -0.688 | -0.5217 | Yes |
| 120 | SHC3 |  |  | 3118 | -0.690 | -0.5173 | Yes |
| 121 | PRR11 |  |  | 3125 | -0.698 | -0.5140 | Yes |
| 122 | HIST1H2AH |  |  | 3139 | -0.716 | -0.5127 | Yes |
| 123 | GAB1 |  |  | 3144 | -0.724 | -0.5086 | Yes |
| 124 | CDK1 |  |  | 3149 | -0.727 | -0.5046 | Yes |
| 125 | S100A16 |  |  | 3155 | -0.736 | -0.5007 | Yes |
| 126 | CDH4 |  |  | 3161 | -0.742 | -0.4968 | Yes |
| 127 | FAT3 |  |  | 3174 | -0.754 | -0.4949 | Yes |
| 128 | SRI |  |  | 3177 | -0.757 | -0.4900 | Yes |
| 129 | USP1 |  |  | 3183 | -0.762 | -0.4860 | Yes |
| 130 | BRCA1 |  |  | 3185 | -0.765 | -0.4807 | Yes |
| 131 | SALL1 |  |  | 3190 | -0.768 | -0.4763 | Yes |
| 132 | TCF12 |  |  | 3191 | -0.769 | -0.4707 | Yes |
| 133 | TCF4 |  |  | 3205 | -0.787 | -0.4689 | Yes |
| 134 | FBXO5 |  |  | 3212 | -0.797 | -0.4649 | Yes |
| 135 | LCORL |  |  | 3256 | -0.859 | -0.4715 | Yes |
| 136 | NLGN1 |  |  | 3259 | -0.865 | -0.4658 | Yes |
| 137 | SOX9 |  |  | 3271 | -0.883 | -0.4627 | Yes |
| 138 | LDLRAD3 |  |  | 3285 | -0.904 | -0.4600 | Yes |
| 139 | SYT11 |  |  | 3286 | -0.904 | -0.4534 | Yes |
| 140 | NKAIN3 |  |  | 3301 | -0.933 | -0.4509 | Yes |
| 141 | TNIK |  |  | 3304 | -0.938 | -0.4446 | Yes |
| 142 | KAT6B |  |  | 3313 | -0.949 | -0.4401 | Yes |
| 143 | WSCD1 |  |  | 3324 | -0.974 | -0.4360 | Yes |
| 144 | GPM6A |  |  | 3328 | -0.976 | -0.4298 | Yes |
| 145 | UBE2T |  |  | 3330 | -0.979 | -0.4230 | Yes |
| 146 | KIF21A |  |  | 3334 | -0.986 | -0.4167 | Yes |
| 147 | CTH |  |  | 3336 | -0.990 | -0.4098 | Yes |
| 148 | ROBO2 |  |  | 3337 | -0.998 | -0.4025 | Yes |
| 149 | GOLM1 |  |  | 3339 | -1.001 | -0.3955 | Yes |
| 150 | EZH2 |  |  | 3342 | -1.015 | -0.3888 | Yes |
| 151 | LSAMP |  |  | 3346 | -1.033 | -0.3821 | Yes |
| 152 | FOXG1 |  |  | 3351 | -1.044 | -0.3757 | Yes |
| 153 | TRIM9 |  |  | 3357 | -1.052 | -0.3696 | Yes |
| 154 | ETV5 |  |  | 3364 | -1.078 | -0.3635 | Yes |
| 155 | NCAPG |  |  | 3370 | -1.098 | -0.3570 | Yes |
| 156 | ZBTB20 |  |  | 3373 | -1.109 | -0.3496 | Yes |
| 157 | RGS12 |  |  | 3375 | -1.111 | -0.3418 | Yes |
| 158 | DPYSL3 |  |  | 3377 | -1.119 | -0.3339 | Yes |
| 159 | NFIB |  |  | 3388 | -1.166 | -0.3284 | Yes |
| 160 | SOX2 |  |  | 3394 | -1.198 | -0.3212 | Yes |
| 161 | TTC28 |  |  | 3397 | -1.215 | -0.3130 | Yes |
| 162 | DCLK2 |  |  | 3400 | -1.237 | -0.3045 | Yes |
| 163 | HIST1H1E |  |  | 3401 | -1.237 | -0.2955 | Yes |
| 164 | LYPD1 |  |  | 3412 | -1.270 | -0.2893 | Yes |
| 165 | NCAM1 |  |  | 3416 | -1.284 | -0.2808 | Yes |
| 166 | MAP2 |  |  | 3422 | -1.305 | -0.2728 | Yes |
| 167 | HIST1H1C |  |  | 3427 | -1.335 | -0.2643 | Yes |
| 168 | KLHDC8A |  |  | 3438 | -1.433 | -0.2569 | Yes |
| 169 | CDH2 |  |  | 3439 | -1.439 | -0.2464 | Yes |
| 170 | MAP3K1 |  |  | 3449 | -1.546 | -0.2378 | Yes |
| 171 | FAM181B |  |  | 3460 | -1.707 | -0.2284 | Yes |
| 172 | TUBB2B |  |  | 3465 | -1.731 | -0.2170 | Yes |
| 173 | HIST1H1D |  |  | 3466 | -1.743 | -0.2043 | Yes |
| 174 | HIST1H1B |  |  | 3467 | -1.770 | -0.1914 | Yes |
| 175 | SOX11 |  |  | 3469 | -1.784 | -0.1787 | Yes |
| 176 | LINC00461 |  |  | 3473 | -1.798 | -0.1665 | Yes |
| 177 | PTN |  |  | 3476 | -1.843 | -0.1537 | Yes |
| 178 | SCRG1 |  |  | 3480 | -1.876 | -0.1410 | Yes |
| 179 | CDCA7L |  |  | 3481 | -1.878 | -0.1273 | Yes |
| 180 | POU3F2 |  |  | 3487 | -2.092 | -0.1135 | Yes |
| 181 | ID4 |  |  | 3488 | -2.097 | -0.0983 | Yes |
| 182 | NFIA |  |  | 3492 | -2.214 | -0.0830 | Yes |
| 183 | HIST1H4C |  |  | 3493 | -2.248 | -0.0667 | Yes |
| 184 | C1orf61 |  |  | 3499 | -2.534 | -0.0497 | Yes |
| 185 | LMO1 |  |  | 3507 | -3.235 | -0.0283 | Yes |
| 186 | PTPRZ1 |  |  | 3510 | -4.002 | 0.0003 | Yes |
Table: GSEA details [plain text format]

  

Fig 2: SINGH\_SOX2\_UPREGULATED\_TRANSCRIPTS\_PMID\_28122245: Random ES distribution      
 Gene set null distribution of ES for **SINGH\_SOX2\_UPREGULATED\_TRANSCRIPTS\_PMID\_28122245**
